# Determinants of students' willingness to accept a measles–mumps–rubella booster vaccination during a mumps outbreak: a cross-sectional study

**DOI:** 10.1186/s12889-015-1899-7

**Published:** 2015-06-20

**Authors:** Hanna W. Donkers, Jeannine L. A. Hautvast, Reinier P. Akkermans, Corien M. Swaan, Wilhelmina L. M. Ruijs, Marlies E. J. L. Hulscher

**Affiliations:** Radboud Institute for Health Sciences, Department of Primary and Community Care, Radboud University Medical Centre, P.O. Box 9101, 117, 6500 HB Nijmegen, The Netherlands; Radboud Institute for Health Sciences, Scientific Institute for Quality of Healthcare (IQ healthcare), Radboud University Medical Centre, Nijmegen, The Netherlands; Public Health Service of Gelderland-Zuid, Nijmegen, The Netherlands; Preparedness and Response Unit, Centre for Infectious Disease Control, National Institute for Public Health and the Environment (RIVM), Bilthoven, The Netherlands

**Keywords:** Mumps outbreak, MMR booster, Vaccination uptake, Willingness to accept vaccination, University students

## Abstract

**Background:**

Despite high vaccination coverage, a mumps outbreak that affected mainly vaccinated university students and their contacts took place in the Netherlands in the period 2009–2012. We presented university students with a hypothetical case in which we offered them a measles, mumps, and rubella (MMR) booster vaccination to control the mumps outbreak. The aim of this study was to get insight into the determinants of university students' willingness to accept this vaccination.

**Methods:**

A questionnaire containing 38 items was developed for the purpose of assessing students' willingness and the psychosocial and social demographic determinants influencing their willingness to accept an MMR booster vaccination. In addition, we explored how organisational characteristics influenced the willingness to be vaccinated. Data were collected at six Dutch universities; a total of 790 students from various faculties were invited to participate. This was a convenience sampling procedure.

**Results:**

687 university students participated (response rate 87.0 %) and 60.4 % of the participants said they would be willing accept the hypothetical MMR booster vaccination. The perceived seriousness of mumps (OR 6.1) was the most important predictor of willingness to accept vaccination. Students who expected the MMR vaccination to be effective and to prevent individual illness and who believed their own vaccination would help stop the epidemic were more likely to be willing than others. The students were more willing to accept vaccination when they perceived that the social norms of significant others and the government favoured vaccination. Organisational characteristics, such as offering vaccination cost free and offering it at the university site, increased students’ willingness.

**Conclusion:**

During a mumps outbreak, university students were generally willing to accept a hypothetical MMR booster vaccination. Risk perception, outcome expectations, perceived social norms, and organisational characteristics should be taken into account in the planning of any vaccination campaign for university students during an outbreak of an infectious disease.

## Background

The Dutch National Immunisation Programme (NIP) has been offered to prevent infectious diseases via vaccination since 1957 [1]. This programme has successfully decreased the incidence of vaccine-preventable diseases including measles and poliomyelitis. The measles, mumps, and rubella (MMR) vaccine that contains the Jeryl Lynn genotype A mumps virus strain was introduced in 1987 and is administered in a two-dose schedule at the ages of 14 months and 9 years. Vaccination coverage rates in the Netherlands are high. Full MMR vaccination coverage of 10-year-old children was 93.9 % in 2013 [2].

Since the introduction of the MMR vaccine, several mumps outbreaks have occurred in the Netherlands. Mumps broke out among vaccinated college students in 2004 [3] and among children living in a community with low vaccination coverage in the period 2007–2008 [4]. The most recent outbreak affected mainly vaccinated university students and their contacts in the period 2009–2012, and it still affects the general population, although to a lesser extent [5, 6]. Presumably, many factors facilitated the transmission of mumps from student to student [3, 5–7]. High rates of social interaction and shared accommodation maintained an environment of close contact. There was a mismatch of the circulating virus (genotype D) with the vaccine virus, effectiveness of the vaccine is reduced (waning immunity) and less natural mumps circulation due to mass vaccination reduced the immunity of the population. Several other countries reported similar outbreaks among vaccinated college students and adolescents [8–11]. The health authorities maintain regular outbreak control aimed at reducing the impact of the outbreak by offering a dose of the MMR vaccine to each student who is not fully vaccinated (i.e., any student previously received only one MMR dose or none at all) [12–14]. The National Institute for Public Health and the Environment (RIVM) coordinates national outbreak responses in the Netherlands. During the most recent mumps outbreak among university students, the responses included raising awareness of mumps, surveillance, outbreak investigations, and providing catch-up vaccination to unvaccinated or incompletely vaccinated students [15, 16].

Since the NIP does not target students [1], knowledge about vaccinating university students as a target population is limited. It is important to get insight into the determinants that influence the students’ decisions about whether to accept vaccination, since high vaccination rates are important in controlling outbreaks and students may sometimes be specific target groups. Vaccination campaigns targeting students during outbreaks could benefit from increased understanding why students would be willing to be vaccinated. The aim of the current study was to get insight into students' willingness to accept vaccination during the most recent outbreak. Regardless of their current vaccination status, we presented the university students with a hypothetical case that offered them MMR booster vaccination to control the mumps outbreak. Our research questions were: How many students would be willing to accept the hypothetical MMR booster vaccination? What student characteristics and what social demographic and psychosocial determinants are related to students’ willingness to accept vaccination? What organisational characteristics influence their willingness?

## Methods

### Sample and procedure

This descriptive study has a cross-sectional design. We developed a questionnaire to assess the determinants of students' willingness to accept a hypothetical MMR booster vaccination. The study population consisted of Dutch-speaking university students. We selected 6 of 13 Dutch universities for diversity in the geographic distribution of the reported mumps cases and subsequent Municipal Health Service information campaigns to raise awareness about mumps.

We based the calculation of the sample size on the precision of the estimated proportion of the outcome variable 'willingness to accept MMR vaccination'. Since no estimate of this proportion was available, we used the value 50 % in the calculation. A total of 267 were needed on the basis of a precision of 6 % and a 95 % confidence interval (CI). We multiplied this number with a design effect (variance inflation factor) to account for clustering between universities. On the basis of an intra-cluster correlation coefficient (ICC) of 0.03, we calculated the total sample size of students to be 600. We aimed to include at least 100 students per university. Two researchers visited each university for 1 day in April or May 2012 and invited all students located in food courts, recreation areas, and libraries to participate. We selected two locations at each university to reach students from various faculties. All students present were individually asked to participate. Verbal consent was obtained from students who were willing to participate. Printed questionnaires were handed out to consenting students, and they were asked to complete them (this took averagely 10 to 15 min). The researchers collected the questionnaires immediately on completion. The students who did not consent were asked for their reasons.

### Questionnaire and variables

The content of the questionnaire was based on the results of our explorative qualitative study [17]. It included organisational characteristics and several concepts derived from the Theory of Planned Behaviour [18] and the Social Cognitive Theory [19]. In this preceding qualitative study, we interviewed 22 unvaccinated students who had recently accepted catch-up MMR vaccination (*n* = 10) or had not (*n* = 12). This study provided extensive information about the broad spectrum of determinants that influenced whether the students accepted the vaccination. The questionnaire was pre-tested and revised in two rounds.

#### Willingness

The primary outcome measure was students' willingness to accept MMR vaccination. After presenting participants with written information about the mumps outbreak, we described a hypothetical case in which an MMR booster vaccination would be offered to all students in order to stop the outbreak. Willingness ('I would definitely accept this MMR booster vaccination') was measured with one item on a four-point scale: “totally agree”, “agree”, “disagree”, “totally disagree”.

#### Student characteristics

We assessed gender, year of study, university city, and study programme.

#### Social demographic determinants

We assessed several determinants: age, country of birth, living situation, and health status. The participants were asked whether they had participated in the NIP as children ('yes', 'no', 'I do not know' and 'partially'). The students' knowledge of the latest mumps outbreak in the Netherlands (‘Do you know there is an ongoing mumps outbreak among students? yes, no’) was measured with one item.

#### Psychosocial determinants

The participants were asked whether they agreed with 19 items designed to assess psychosocial determinants of their willingness to be vaccinated. All items were measured on a four-point scale: ‘totally agree’, ‘agree’, ‘disagree’, ‘totally disagree’. *Attitude toward vaccination* was assessed with three items (e.g., 'I generally do not object to vaccination'). *Risk perception* was measured with four items about perceived severity and perceived susceptibility (e.g., 'Mumps can have serious consequences for my own health' and 'I think I have a high risk of catching mumps'). *Outcome expectations* were measured with nine items about perceived benefits and anticipated regret (e.g., 'I think vaccination decreases my chances of getting mumps' and 'If the epidemic turns out not to be severe, I would regret getting vaccinated'). The *perceived social norm* was measured with three items (e.g., 'I would discuss the advice to get vaccinated with significant others').

#### Organisational characteristics

The questionnaire contained eight questions about organisational characteristics to find out which ones influenced willingness to accept the hypothetical MMR booster. Questions about organisational characteristics that might influence the accessibility of getting vaccination included the vaccination venue (e.g., a university site or a location of the Municipal Health Service), price (e.g., cost free or 20 euros), invitation type (e.g., personal or via a newspaper), time constraints (a busy study period), and a financial incentive (a small reward after vaccination). Responses to these items were recorded on a four-point scale: ‘yes, certainly’, ‘yes, probably’, ‘no, probably not’, ‘no, certainly not’.

### Data analyses

We used SPSS for Windows Release 19 for the descriptive statistics to describe the participants' social demographic variables. The scores for the psychosocial determinants and the item measuring willingness (the primary dependent variable) were converted to dichotomous scores (agree – disagree). Spearman correlation coefficients were computed since our data were not normally distributed. Only the determinants that showed statistically significant associations with willingness to be vaccinated (*p* < 0.05) and with correlation *r* ≥ 0.25 were entered in a multilevel logistic regression analysis [20, 21]. Because of the hierarchical structure of our study (students nested within universities), we based these analyses on the logistic mixed effect model (PROC GLIMMIX in SAS V9.2). Both fixed and random effects can be analysed in this model. We used a model with a random intercept; all other variables were fixed. We considered *p* < 0.05 statistically significant. We performed descriptive statistics for the eight organisational characteristics.

## Results

### Characteristics of the respondents

A total of 687 Dutch university students completed the questionnaire. The number of questionnaires returned from the universities ranged from 102 to 121. The total response rate was 87.0 %, ranging from 80.8 % to 95.2 % per university. The 103 non-consenting students all reported refusing to participate due to self-reported time constraints. None of the students who refused to participate mentioned religious or philosophical objections to vaccination.

Of all the participants, 50.2 % were male. Students from all study programmes and years participated. The mean age of the participants was 21.3 years (SD 2.7 years). Most of the respondents, 87.3 %, had Dutch citizenship, and 72.7 % of them lived away from home. Most students reported good health: 96.8 % assessed their health as excellent, very good, or good. Most of the participants, 90.5 %, had received all National Immunisation Programme (NIP) vaccinations during childhood; 2.2 % did not participate; 1.8 % had received at least one vaccination; and 5.5 % had no knowledge of their vaccination status. Of all participants, 38.7 % knew there was an ongoing mumps outbreak among university students in the Netherlands, with a city range from 18.5 % (Eindhoven) to 61.3 % (Nijmegen).

### Vaccination willingness

A total of 408 participants (60.4 %) were willing to accept the hypothetical MMR booster vaccination.

### Social demographic determinants

Of the 408 students who were willing to accept the MMR vaccine, 48.8 % were male and 51.2 % were female (Table 1). The willingness of the students from different years of study ranged from 50.4 % (third-year students) to 65.5 % (first-year students). A greater proportion of students studying in Delft were willing to accept vaccination (75.2 %) than in other cities. Willingness varied among the study programmes as well; the people who studied linguistics, history, or arts were least inclined to accept vaccination (37.8%).

**Table 1:** Willingness and student characteristics of a sample of Dutch university students

Item	Characteristic	*Study subjects* (%)	Willing to be vaccinated (%)
		*n=687*	*n=408/676*
Gender	Male	345 (50.2)	199 (48.8)
	Female	342 (49.8)	209 (51.2)
		*n=683*	*n=404/672*
Year of study	1	260 (38.1)	167 (65.5)
	2	147 (21.5)	92 (63.4)
	3	134 (19.6)	66 (50.4)
	≥4	142 (20.8)	79 (56.0)
		*n=687*	*n=408/676*
University city	Nijmegen	119 (17.3)	69 (58.0)
	Eindhoven	119 (17.3)	67 (56.3)
	Utrecht	122 (17.8)	68 (58.1)
	Delft	102 (14.8)	76 (75.2)
	Amsterdam	121 (17.6)	61 (51.7)
	Maastricht	104 (15.1)	67 (65.7)
		*n=682*	*n=406/671*
Study programme^a^	Social sciences, Business, Law	226 (33.1)	129 (58.1)
	Technique, Industry, Engineering	179 (26.2)	113 (63.5)
	Healthcare, and Welfare	141 (20.7)	82 (59.0)
	Physics Mathematics, IT	55 (8.1)	42 (76.4)
	Linguistics, History, Arts	39 (5.7)	14 (37.8)
	Other	42 (6.2)	26 (65.0)

^a^based on the International Standard Classification of Education (ISCED) division [35]

Spearman correlations with vaccination willingness were calculated for student characteristics and social demographic variables including gender, year of study, university city, study programme, living situation, health status, NIP participation, and knowledge of the epidemic. Only the year of study correlated statistically significantly with willingness (*r* < 0.25). These variables were not included in further analyses because of the weak correlation.

### Psychosocial determinants

Table 2 presents the results from the descriptive statistics of the dichotomous psychosocial determinants and the Spearman correlations of their relation with the willingness to be vaccinated. All items were associated with willingness (*p* ≤ 0.05). Correlations with willingness (*r* ≥ 0.25) were found for nine psychosocial variables related to attitude, risk perception, outcome expectations, and perceived social norms.

**Table 2:** Descriptive statistics and Spearman correlation of psychosocial determinants and willingness to be vaccinated

Questionnaire statement	Concept	Agreement *n* (%)	Spearman’s *rho*	*Significance*
I have no objections to vaccination in general	Attitude 1	646 (94.6)	0.209	<0.0001
I sometimes doubt the safety of vaccinations	Attitude 2	229 (33.4)	−0.156	<0.0001
Vaccinating all students is costly and therefore not desirable	Attitude 3	171 (25.1)	−0.267	<0.0001
I think mumps can have serious consequences for my own health	Risk perception 1	483 (70.8)	0.548	<0.0001
I think I have a high risk of catching mumps	Risk perception 2	108 (15.9)	0.208	<0.0001
It is better to live through mumps, so I would not get vaccinated	Risk perception 3	52 (7.7)	−0.158	<0.0001
I do not think mumps is serious for me, so I would not get vaccinated	Risk perception 4	180 (26.6)	−0.531	<0.0001
Possible side effects of vaccination stop me from getting vaccinated	Outcome expectations 1	281 (41.6)	−0.185	<0.0001
I doubt the safety of the MMR vaccine	Outcome expectations 2	91 (13.5)	−0.134	0.010
The MMR vaccination does not work well, so I would not get vaccinated	Outcome expectations 3	95(14.4)	−0.101	0.010
I would accept vaccination to prevent myself from becoming ill	Outcome expectations 4	530 (77.9)	0.515	<0.0001
As a result of intensive student contacts, mumps cannot be stopped	Outcome expectations 5	210 (31.0)	−0.150	<0.0001
I think vaccination decreases my chances of getting mumps	Outcome expectations 6	624 (91.4)	0.275	<0.0001
I would accept vaccination if it were offered as a means of stopping the epidemic	Outcome expectations 7	546 (79.8)	0.412	<0.0001
If I decide not to get vaccinated and I catch the mumps, I would regret my decision	Outcome expectations 8	453 (66.9)	0.193	<0.0001
If the epidemic turned out not to be severe, I would regret getting vaccinated	Outcome expectations 9	176 (26.0)	−0.272	<0.0001
I would discuss the advice to get vaccinated with significant others	Social norm 1	556 (81.8)	−0.100	0.009
Significant others think it is important for me to get vaccinated	Social norm 2	453 (67.8)	0.339	<0.0001
The government advises vaccination, so I would accept it	Social norm 3	341 (50.4)	0.385	<0.0001

These nine determinants were analysed in a multilevel logistic regression model. Table 3 presents the psychosocial determinants showing a statistically significant (*p* < 0.05) relation to students' willingness to accept MMR booster vaccination in the multilevel analysis. These seven variables explained 58 % of the variance in willingness (R^2^). One item relating to the perceived seriousness of mumps (risk perception 1) showed the most powerful influence on willingness to be vaccinated (OR 6.1; CI 95 % 3.54–10.35) with a higher risk perception that resulted in greater willingness. Students who believed mumps was not serious for them were less likely to accept vaccination (OR 0.25; CI 95 % 0.14–0.43). Students who expected vaccination to prevent their own illness (outcome expectation 4; OR 2.8, CI 95 % 1.47–5.37) and those who expected that individual vaccination would help stop the epidemic (outcome expectation 7; OR 2.5, CI 95 % 1.32–4.67) were more likely to be willing. Expecting to regret vaccination if the epidemic turned out not to be severe (outcome expectation 9; OR 0.5, 95 % CI 0.33–0.90) resulted in less willingness. Perception of the social norm influenced willingness as well. If significant others thought vaccination was important (social norm 2; OR 1.7, 95 % CI 1.01–2.73) or if the government advised vaccination (social norm 3; OR 2.2, 95 % CI 1.40–3.60), students were more likely to accept vaccination.

**Table 3:** Multilevel multivariate logistic regression analysis of significant determinants willingness to be vaccinated

Questionnaire item	Concept	Odds ratio	95 % CI for OR Lower upper		*t Value*	Significance
I think mumps can have serious consequences for my health	Risk perception 1	6.06	3.54	10.35	6.59	<0.0001
I do not think mumps is serious for me, so I would not get vaccinated	Risk perception 4	.25	.14	.43	−4.93	<0.0001
I would accept vaccination to prevent myself from becoming ill	Outcome expectation 4	2.80	1.47	5.37	3.12	0.0019
I would accept vaccination if it were offered as a means of stopping the epidemic	Outcome expectation 7	2.48	1.32	4.67	2.83	0.0049
If the epidemic turned out not to be severe, I would regret getting vaccinated	Outcome expectation 9	.54	.33	.90	−2.35	0.0189
Significant others think it is important for me to get vaccinated	Social norm 2	1.66	1.01	2.73	2.00	0.0454
The government advises vaccination, so I would accept it	Social norm 3	2.24	1.40	3.60	3.35	0.0009

### Organisational characteristics

To determine which organisational characteristics influence their willingness, we presented all 408 students who were initially willing to accept the MMR vaccine with statements about accepting vaccination in various situations (Table 4). Almost all of the students who were initially willing (97.8 %) were willing to accept the vaccination if it was offered cost free. If the students had to pay for vaccination, 58.4 % of all students who were initially willing would still accept it. The vaccination venue also influenced the students’ willingness. If the venue was the university site, 94.3 % of the students with initial willingness would still accept vaccination compared to 82.6 % when the venue was at a local Municipal Health Service. The number of students willing to accept vaccination decreased if they had time constraints such as a busy study period (68.9 %). Further, 91.8 % of the students who were initially willing would accept vaccination if they received a small reward afterwards. Willingness was influenced by the type of invitation: 94.0 % of the students invited in person would get vaccinated, whereas only 60.6 % would get vaccinated if they were invited by a general call in a newspaper or on the internet.

**Table 4:** Descriptive statistics of organisational characteristics and willingness to be vaccinated among students who were initially willing^a^

Questionnaire item	Agreement *n* (%)
I would accept vaccination even if I had to pay for it myself (±20 euros)	237 (58.4)
I would accept vaccination if it were offered free of charge	397 (97.8)
I would accept vaccination if it were offered at a university site	383 (94.3)
I would accept vaccination if it were offered at the local Municipal Health Service	336 (82.6)
I would accept vaccination even if I were very busy studying	279 (68.9)
I would accept vaccination if I received a small reward for it	371 (91.8)
I would accept vaccination if I received a personal invitation	378 (94.0)
I would accept vaccination if students were invited in newspaper ads or on the internet	246 (60.6)

^a^The total number of students who were initially willing was 408

## Discussion

Our study shows that 60.4 % of Dutch university students were willing to be vaccinated in the hypothetical case of an offer of an MMR booster vaccination to control a mumps outbreak. A greater proportion of respondents based in Delft were willing to accept vaccination (75.2 %) than in the other cities. This was probably due to the large clusters of mumps cases reported from Delft in 2010 [6, 7]. The overall proportion of students who were willing in our study is consistent with the findings of a similar study among university students in the UK [22].

Risk perception was the most important factor associated with willingness to be vaccinated. Over 70 % of the students believed that mumps could have serious consequences for their own health, and students who considered mumps to be serious were more likely to accept MMR vaccination. This finding is consistent with psychosocial theories about preventive behaviours [23, 24] that indicate that perceived seriousness of a health threat induces increased willingness to accept preventive behavioural measures. According to these theories, knowledge of the existence of a health risk, such as a mumps outbreak, is not enough to elicit preventive behaviours such as obtaining vaccination. This is in line with our results, since knowledge did not correlate significantly with willingness. Moreover, to become motivated to protect themselves and accept vaccination, students would have to realize that they are personally at risk. However, in our study, only 16 % of the respondents believed that they had a high risk of catching mumps. This *perceived susceptibility* was not strongly correlated with vaccination willingness. Similarly, a study [25] of college students' willingness to accept the novel H1N1 vaccine did not show perceived susceptibility to be a statistically significant predictor. Thus, the perceived seriousness of the disease seems to be a more important determinant of getting vaccination than perceived susceptibility.

The perceived benefits of the vaccination (*outcome expectation*) had a powerful influence on willingness: students were more likely to accept vaccination if they believed that vaccination would reduce their risks of catching mumps and if they believed that accepting vaccination would help end the epidemic. Similar, a study in England that involved university students and staff during a mumps outbreak and a consecutive immunisation programme [26] also shows that perceived individual benefit is an important factor in the individual’s decision to accept vaccination. This can be taken into account in the messages that are part of an information campaign targeting students during an outbreak.

*Perceived social norm*, or significant others’ approval of behaviour, had a substantial influence on willingness to be vaccinated in our study. If significant others thought vaccination is important or if the government advised vaccination, then the students were more likely to accept vaccination. Other studies show that social norms are important factors in decisions about vaccination as well. Hamilton-West [26] reports that actual peer decision to be the most important predictor of vaccination acceptance; American students’ willingness to be vaccinated during the H1N1 pandemic was statistically significantly associated with the attitudes of people in their social networks [27]. Therefore, vaccination campaigns targeting students during outbreaks might benefit from using fellow students as role models to promote vaccination.

This study shows that *organisational characteristics* can negatively influence students’ willingness and thus become barriers to their vaccination. Accessibility factors such as the price students have to pay for vaccination, the location where vaccination is offered, and the manner of inviting students resulted in variation of the proportions of students who were willing. Similarly to our findings, Canadian research about hepatitis B immunisation of healthcare students [28] and research into factors affecting the immunisation status of American students [29] shows the need for a low-cost vaccine, since payment can be a barrier to students being vaccinated. Another method to increase vaccination to consider is offering a reward; however, this might lead to ethical issues and therefore requires thorough deliberation before implementation in practice.

Social demographic determinants such as gender, study programme, study year, living situation, health status, knowledge of the latest mumps outbreak, and acceptance of NIP vaccinations in childhood were not directly related to willingness to be vaccinated. These findings are in accordance with earlier research into determinants of Dutch parents' intentions to vaccinate their children [30].

A strength of this study is the large sample of respondents who participated because of our personally visiting universities and asking students to participate. However, this procedure for convenience sampling may limit the generalisation of the results. Only students present in public areas of the selected universities were approached, which might have introduced a selection bias. Nevertheless, the social demographics of the students in this sample did not differ from those of average Dutch students [31]. Our qualitative study among incompletely vaccinated students that we conducted during the mumps outbreak in the Netherlands has revealed critical vaccination attitudes concerning side effects and vaccine effectiveness [17]. Although 103 students refused to participate due to self-reported time constraints, there is no reason to believe that this attrition is selective with regard to students with critical vaccination attitudes. In general, the students were not aware of the study subject before they refused to participate and none of them said that they declined to participate because of religious or philosophical objections to vaccination.

Even though our study shows that students were generally willing to accept the MMR booster vaccination during an outbreak, there is no conclusive evidence that routine administration of a third dose of MMR vaccine is an effective means of outbreak control [32–34]. Our study included students regardless of their vaccination status. This limits generalisation to the student population targeted recently during the mumps outbreak, since only incompletely vaccinated students were actually offered vaccination. Future research aimed at identifying determinants that influence the behaviours of incompletely vaccinated students who did not respond to the vaccination offer could therefore be valuable.

## Conclusions

This study provided valuable insight into students’ willingness to be vaccinated and into related determinants when presented a hypothetical scenario in which an MMR booster vaccination was offered as a means of controlling an MMR outbreak. Risk perception, outcome expectations, perceived social norms, and organisational characteristics should be taken into account in the planning of an actual vaccination campaign for students during an infectious disease outbreak.

### Ethical considerations

The research ethics committee of Radboud University Medical Centre of Nijmegen approved this study: CMO number 2011/411. Participation in this study was voluntary, and the respondents could withdraw from participation at any time. We obtained the respondents’ implicit informed consent by providing them with oral and written explanations of the nature of the study before they answered the questionnaire.
